# Notes of the Week

**Published:** 1921-07-09

**Authors:** 


### NOTES OF THE WEEK.

### THE STRENGTH OF PROVINCIAL HOSPITALS.

The report of the British Hospitals Association presented at the annual meeting at Manchester shows a record of activity which cannot fail to make the influence of the Association felt in shaping the new plans and policies which it is inevitable hospitals must be prepared to adopt, if the voluntary system is to be maintained, as all are now agreed it must be. The Regional Committees which have been formed during the past year have proved themselves a valuable link in the co-ordination of the work. Under the new rules of the Association the Council will in time consist almost entirely of representatives of Regional Committees, so that its judgment and decisions may be taken generally to represent the considered opinion of the whole of the voluntary hospitals of the country. By this means, too, public opinion throughout the country should be kept informed of the work the hospitals are doing, their needs and claims. Too much importance cannot be attached to the opportunities now afforded to the Regional Committees, and Sir Arthur Stanley's call to them to rise to the occasion should bear fruit. We are asked to state that the meeting of the London Regional Committee has been postponed from July 12 to July 26, so as to be in a position to consider the report of Lord Hambleden's Committee, and also that of a deputation on salaries to the College of Nursing arranged for July 19.

### HOSPITAL BENEFIT AND APPROVED SOCIETIES.

The British Hospitals Association has been in communication with the Approved Societies, and one, the National Deposit Friendly Society, has agreed to provide payment at the rate of one guinea per week in respect of hospital treatment of its members; the proposal is to open a fund with the sum of £100,000, to which will be added interest and the proportion of benefit receivable from the Treasury, out of which fund the Society will make payment for treatment either to a hospital direct or to any body or association representing hospitals. Any unexpended portion of the fund at the expiration of five years from July 1921 shall be brought into account at the next valuation. It is also provided that not more than one-fifth of the total sum available for the forthcoming five years shall be expended in any one year, except that if in any yearly period the amount expended be less than that available the surplus may be expended in any subsequent year. The payments under this scheme are to begin from July 4, 1921. It is to be hoped that with the lead of the National Deposit Friendly Society before them the other Approved Societies will be prompt in announcing their intentions; the delay may to some extent be due to the fact that the largest organisations have not yet received their valuation reports. Lord Hambleden's Committee of the British Hospitals Association, which was appointed to deal with this question of contributions from Approved Societies, is, however, meeting on July 12, and it is anticipated that it will have certain progress to report to the London Regional Committee. It will be seen that at the annual meeting at Manchester it was resolved to ask for a block grant if possible in preference to a *per capita* payment.

### IRELAND AND THE CAVE COMMISSION.

The Dublin hospitals have expressed dismay that Ireland is not included in the grant of £500,000 which the Government has agreed to give to the voluntary hospitals. At the beginning of the Commission's report, however, it is expressly stated that "we understand that the Irish hospitals are not included in our reference," although it is fair to say that the reference itself mentioned "the present financial position of the voluntary hospitals" without precise definition. The Chairman of the Board of Superintendence of Dublin Hospitals, Sir Lambert Ormsby, recalled that no distinction against Ireland had been made in the administration of the Prince of Wales's Fund. When Capt. Redmond asked Sir Alfred Mond why Ireland was excluded, the Minister of Health replied that the Irish local Parliaments could make grants. It is pointed out that, while this may be all right for Northern Ireland, it is at present uncertain if and when the Southern Parliament will meet. In our view, if the Commission's exclusion of Ireland from the terms of its reference was arbitrary, yet the grant promised by the Government is so small that, even with the exclusion of the Irish hospitals, each institution stands to receive very little. As a matter of fact, the Irish hospitals already receive an annual grant of £16,000 from the Government.

### COUNTY COUNCILS AND HOSPITAL SUBSCRIPTIONS.

The Finance Committee of the East Suffolk County Council lately asked the Ministry of Health if the Council, as an employer of labour, may contribute like other employers the same amount as that given by its staff to the East Suffolk and Ipswich Hospital. The following reply has been received: "On further consideration, it seems to the Ministry that the County Council can only subscribe for the benefit of their workmen if it were arranged with the workmen that the subscriptions are to be paid on their behalf, and as being part of their wages." A member's remark on this reply was that he did not mind how the contribution was paid so long as the County Council came into line with the agricultural employers of the country. But these, presumably, contribute a sum equal to that subscribed by their employers; whereas the Ministry's letter seems to say that the Council's employees may contribute out of their wages, but that the Council itself cannot add to the amount.

### FREE OF DEBT FROM ITS FOUNDATION.

Mr. Carmichael Thomas, Chairman of the Central London Throat, Nose, and Ear Hospital, has given to the *Pall Mall and Globe* his views upon the Report of the Cave Commission. Since he is resolutely in favour of the recommendations, his most interesting remarks concern the system by which his hospital, since its foundation in 1874, has been "run for the last forty-seven years free of debt" and saved something each year towards the necessary extension of the building. Mr. Thomas attributes this probably unique record to the fact that those patients in a position to pay something have always been invited to contribute, so that he claims that the other hospitals are now following the example set by the Central London Ear Hospital nearly fifty years ago. In 1920 the average proportion of in-patients who contributed was 60 per cent.; 40 per cent. were treated free; and the average amount contributed by in-patients was £1 3s. 5d. for the whole of their stay. The record is a remarkable one, but the circumstances of hospitals vary so much that it is doubtful if these voluntary contributions by patients suffice to explain it. None the less, the time to emphasise that record is now, and Mr. Carmichael Thomas and the Secretary, Mr. R. Kershaw, may justly be proud to proclaim it.

### A SIGHT FOR HOSPITAL VISITORS.

A party of fifty, representing the Committee and workers of the Camberwell, Brixton, and Walworth branch of the Metropolitan Hospital Saturday Fund, has made a special visit to King's College Hospital. Received by Mr. R. J. Coles, the Secretary, and Miss Willcox, R.R.C., the matron, the party made a tour of the hospital, and saw not only the active departments, but the closed wards. Mr. W. Wescott, the President, and Mr. W. Appleby, the Chairman of the branch, promised to do all they could to interest their friends in the needs of the hospital, which are also the needs of the neighbourhood and the sick. When replying to the thanks of the visitors, Mr. Coles argued strongly in favour of systematic contributions of 2d. a week. He would like South London to show that such a scheme can be worked; if only 150,000 people gave this sum the closed wards in King's College Hospital could be reopened. He therefore advocated a series of meetings in the autumn to explain and support the scheme in South London. Mr. Coles's unflinching fight for this principle in London, where so many believe it cannot be successfully maintained, deserves every acknowledgment. In our view the meetings proposed should not be general, but preceded by discussions with the employers, so that the example of those who first consent may give the meetings a send-off. If the heads of the businesses with numerous retail shops can be interested, the seeds of the scheme will be scattered all over London.

### THE RIGHT SPIRIT.

Just as a Bristol firm has lately agreed to pay the maintenance charge of its employees when in hospital, in order to encourage them to continue and increase their weekly contributions, so a Newcastle firm, whose chief and staff likewise subscribe through the Saturday Fund to the Royal Victoria Infirmary there, has sent a donation of three guineas for the treatment of a workman who met with an accident and was kept in the hospital for about a week. It is pointed out that the accident happened out of the man's working hours, and that the firm and its employees are already contributors. The spirit shown is admirable, and may also encourage the Newcastle Royal Infirmary to consider favourably contributions from patients, which have not yet been adopted there. The hospital's financial position is very serious, notwithstanding the workmen's contributions.

### THE PRINCE AND THE HOSPITALS.

H.R.H. the Prince of Wales (President) will visit Guy's Hospital on Friday afternoon, July 15, to unveil the War Memorial and to open the new massage building. On July 7, as we go to press, H.M. the King is lending St. James's Palace for a garden-party on behalf of St. Thomas's Hospital, and is doing the same on July 13 for the League of Mercy, at which latter function the Prince of Wales has intimated his intention to be present. Princess Alice, Countess of Athlone, who is Lady Grand President of the League, will also attend. On July 27 the Prince of Wales will lay the foundation-stone of the new Nurses' Home of the Royal Victoria Hospital, Folkestone.

### THREAT TO BRADFORD INFIRMARY SITE.

The authorities of the Bradford Royal Infirmary have raised an objection to the Corporation's proposal to divert Duckworth Lane, because the diversion would cut a corner of the Field House estate, which has been purchased for the new infirmary. Though no building has begun, the governors of the infirmary contend that their site would be interfered with by the Corporation's scheme. The architect, Mr. W. A. Pite, is to discuss the matter with the city surveyor, in the presence of Mr. G. L. Pepler, the Inspector of the Ministry of Health, who is holding an inquiry into the schemes which the Corporation is seeking permission to develop.

### A CHAIRMAN ON PRICES.

Mr. H. Huntsman, who presided over the quarterly meeting of governors of the Sheffield Royal Infirmary, made some remarks upon the present state of prices. He said that a sensible reduction of expenses has taken place during the past quarter. Food, milk, and medical supplies have been affected. The cost of medical supplies has been extortionate. There has been a considerable improvement, but he will not say that medical supplies have yet reached a reasonable figure. In regard to coal, they still have supplies for two or three weeks. Some of the outcrop workers sent to them gifts of about one hundred tons. In the South of England the price of milk and butter threatens to rise in consequence of the drought. It is to be hoped, however, that this is only a temporary interruption of the decline in prices.

